# Obstructions in the Larynx and Trachea

**Published:** 1882-06

**Authors:** E. Fletcher Ingals

**Affiliations:** Professor of Diseases of the Throat and Chest, Woman’s Medical College of Chicago; Physician and Surgeon for Diseases of the Throat and Chest, Central Free Dispensary, etc.


FOR CONTENTS OF THIS NUMBER SEE PAGE 432.

THE

### Independent Practitioner.

Vol. III.——*June*, 1882.——No. 6.

### ORIGINAL COMMUNICATIONS.

*We are not responsible for the opinions expressed by contributors.*

ARTICLE I.

### OBSTRUCTIONS IN THE LARYNX AND TRACHEA.

BY E. FLETCHER INGALS, A.M., M.D.,
Professor of Diseases of the Throat and Chest, Woman's Medical College of Chicago; Physician and Surgeon for Diseases of the Throat and Chest, Central Free Dispensary, etc.

[Read before the Illinois State Medical Society, May, 1882.]

In October last a boy eleven years of age was brought to me from Waukegan, suffering from severe dyspnœa caused by a foreign body in the air-passages.

I learned that a month previously, while cracking a hickory-nut with his teeth, the boy was made to laugh, when a piece of the shell was drawn into his larynx. Severe cough and dyspnœa resulted, but after a few days the latter nearly subsided, though the cough remained. Thus he continued for three weeks. Then the dyspnœa again became troublesome, and it steadily increased up to the time when he was brought to my clinic, at the Central Free Dispensary, one month after the accident. I found the patient very hoarse and greatly troubled for breath.

A laryngoscopic examination was made, but the boy's timidity prevented me from obtaining a satisfactory view of the lower part of the larynx, though I obtained a glimpse of the glottis, which enabled me to determine that no body of any considerable size was lodged between or above the vocal cords.

As the changes which had taken place in the boy's symptoms during the last few days indicated that the foreign body was not likely to change its position within a few hours, I did not press the examination further, but directed the patient's mother to bring him to my office the following morning; but, to avoid accident, I cautioned her to send for me at once should any serious dyspnœa occur during the night.

This was at five o'clock in the afternoon. Four hours later I was summoned, by telephone, to see the patient immediately at his lodgings.

I found him in a small room so damp and chilly as to endanger the life even of a perfectly healthy child. There was no place for a fire, the ceiling was wet from recent rains, and in some places the carpet was soaked with water.

The child was laboring for breath, the soft parts falling in with each inspiration, and suffocation was certain to ensue within a short time unless relief could be obtained. I at once set about finding a better room, which was soon secured in a neighboring hotel.

Doctors R. S. Hall, and B. W. Griffin came to my assistance, and as the patient's condition precluded the idea of further laryngscopic examination, no delay being permissible, we made immediate preparations to open the trachea.

The body being fixed, there could be no objection to an anæsthetic; therefore chloroform was used, which is not, like ether, liable to explode in consequence of ignition from the lights.

After dividing the skin with my scalpel, I attempted to reach the trachea by means of the galvano-cautery, hoping thus to avoid accidental hemorrhage, which might occur when operating in a poor light; but the poor light, and my assistant's difficulty in controlling my battery, with which he was unfamiliar, rendered this part of the operation unsatisfactory. Meantime the patient stopped breathing. I then threw aside the galvano-cautery and speedily worked my way down to the trachea and opened it with the handle and blade of my scalpel. Artificial respiration was then instituted and continued a few minutes until natural respiration was established.

I then introduced my tracheal forceps, and having found the trachea clear, turned them upward, when I found the shell firmly embedded in the larynx just below the vocal cords. It was so firmly held that several times the forceps slipped off before I succeeded in extracting it. The piece of shell was of triangular form, with sharp borders and angles. It measured three-fourths of an inch in its longest diameter, by half an inch across its base. The shape would allow of its passing through the glottis lengthwise, with its sides anteriorly and posteriorly, and while it remained in this position in the trachea it would not greatly obstruct the calibre of the tube ; but, soon as it became turned, very little space would be allowed for the passage of air. If it had been short enough so that it could have turned horizontally across the trachea, it would very likely have caused fatal suffocation during some of the paroxysms of cough which occurred soon after the accident.

From the history I concluded that the shell had at first fallen into the trachea, where it had remained three weeks, and that then it had been coughed up into the larynx, fortunately lodging edgewise. The night before I saw the child he nearly suffocated, either from change in the position of the shell, or from spasm and swelling of the larynx, induced by the irritation which it set up and his bad surroundings.

After the operation I introduced a tracheal tube and allowed it to remain three days, until the inflammation of the larynx had subsided.

Broncho-pneumonia supervened, the temperature rising four or five degrees, but by the end of the fifth day the untoward symptoms began to disappear, and the child subsequently made a speedy recovery. In eight days after the operation the wound was closed so that no air escaped through it, and the patient left the city. I saw him about four weeks later. The wound was entirely healed and the voice perfect.

A few weeks after treating this case I was asked by my friend, Dr. W. S. Dorland, to see a child eighteen months old, who had drawn a bit of bone into the larynx the previous evening.

A neighboring physician had been summoned when the accident first occurred, but as there was but little dyspnœa and there seemed no immediate danger, Dr. Dorland, who was the family physician, had not been called until the next morning. He found the child suffering from a little dyspnœa, but no hoarseness, and yielding slight signs of obstruction in the air-passages.

The doctor told the friends he wished to bring me in to see the case, and we arranged to go at four o'clock in the afternoon of the same day, which was the earliest convenient hour.

Just as we were starting we received a telephone message that the child was in a convulsion. We drove rapidly to the house, but did not arrive until the child had, to all appearance, been dead for half an hour ; indeed, it had ceased to breathe before the messenger left the house to telephone to us. An examination showed that the heart had ceased to beat, and the appearance of the body precluded the idea of resuscitation.

A post-mortem examination was made the next day, and the bone was found firmly fixed in the sub-glottic portion of the larynx, where it had caused considerable laceration and ulceration of the mucous membrane, showing that it must have been in that position for several hours. Upon examining the heart, Dr. Dorland found a firm, white, fibrinous clot, extending from the left ventricle through the aortic valves, and a few small, dark clots in the right side of the heart.

Each of these cases illustrates the danger of delaying the operation for the removal of foreign bodies from the air-passages.

The autopsy in the latter case revealed one of the dangers from obstruction of the air-passages which has not been appreciated by the profession, viz., the formation of a heart-clot as a result of the obstructed respiration. This seems to me a matter of very great importance, particularly when the obstruction results from pseudo-membranous deposits.

Tracheotomy, when performed for the relief of patients suffering from diphtheritic croup, is much more successful if done early; different reahave been assigned for this by those who favor and those who oppose the operation.

The former claim that when the operation is delayed the blood becomes so charged with carbonic acid that its elimination is often impossible after a free passage has been made, and that the depression caused by the obstruction favors the more rapid deposit of false membrane, so that only a small percentage can recover of those operated upon after suffocation becomes imminent.

On the other hand, those who are not in favor of tracheotomy in this condition, claim that the greater percentage of favorable results in those who are operated upon early is mainly due to the mildness of the attack, and they infer that most of the favorable cases would have recovered without the operation.

Though there is much plausibility in the reasons assigned by both parties for the greater percentage of recoveries after early operations, I believe that in many cases an early operation saves life by preventing the formation of a fatal heart-clot.

This little child died in twenty-two hours after the accident, and though she had previously been perfectly healthy, the autopsy revealed a firm ante-mortem heart-clot. It must not be forgotten that this patient had experienced but comparatively little dyspnœa, excepting during three or four short paroxysms which occurred at irregular intervals after the accident.

If a firm clot can be formed under such circumstances, how much more likely is it to be formed in patients suffering from the depressing effects of diphtheria when the obstruction of the larynx throws an increased burden on the already weakened heart?

In a case of diphtheritic croup upon which I recently performed tracheotomy for Dr. David Dodge, I examined the child's heart at half-past ten in the forenoon, and found it beating rhythmically, without abnormal sounds. I had seen the child an hour and a half previously, and had thought the operation advisable; but at this time the breathing was easier, so that we concluded to wait until the afternoon, hoping that improvement might take place.

We met at five o'clock in the afternoon of the same day, and found the child so much worse that it seemed impossible for it to survive more than twenty-four hours at most, unless the dyspnœa could be relieved. I then opened the trachea and inserted a tube. Shortly after the operation was completed I placed my ear over the base of the heart and discovered loud murmurs, which I feared came from a heart-clot; but subsequently the sounds were so changed that I concluded they came mostly from the pericardium. These sounds gradually became less distinct and finally disappeared about the fourth day, without other evidence of pericarditis; therefore, considering the character of the murmur as first heard, I still suspect that there was also a clot, which finally softened and was absorbed. The child made a complete recovery in about two weeks.

In cases of foreign bodies in the air-passages the rule should be to operate as soon as possible, if the inversion method does not succeed in removing them.

In membranous croup, especially if of diphtheritic origin, the operation in order to be most successful must be done early, before the dyspnœa has become marked; but, even after there is great obstruction, the operation should be recommended, excepting in the most malignant cases, for it will occasionally save desperate cases, and, even though it should fail to save the patient's life, it will save him much distress.

